# Persistent Endothelial Lung Damage and Impaired Diffusion Capacity in Long COVID

**DOI:** 10.3390/jpm13091351

**Published:** 2023-08-31

**Authors:** Andreas T. Asimakos, Alice G. Vassiliou, Chrysi Keskinidou, Stavroula Spetsioti, Archontoula Antonoglou, Charikleia S. Vrettou, Panagiotis Mourelatos, Aristidis Diamantopoulos, Maria Pratikaki, Nikolaos Athanasiou, Edison Jahaj, Parisis Gallos, Anastasia Kotanidou, Ioanna Dimopoulou, Stylianos E. Orfanos, Paraskevi Katsaounou

**Affiliations:** 1 First Department of Critical Care Medicine & Pulmonary Services, School of Medicine, National and Kapodistrian University of Athens, Evangelismos Hospital, 106 76 Athens, Greece; silverakos@gmail.com (A.T.A.); alvass75@gmail.com (A.G.V.); chrysakes29@gmail.com (C.K.); roula_spe@hotmail.com (S.S.); arxoantonoglou@gmail.com (A.A.); vrettou@hotmail.com (C.S.V.); nikolaosathanasiou14@gmail.com (N.A.); edison.jahaj@gmail.com (E.J.); akotanid@med.uoa.gr (A.K.); idimo@otenet.gr (I.D.); stylianosorfanosuoa@gmail.com (S.E.O.); 2 Department of Endocrinology Diabetes and Metabolism, National Expertise Center for Rare Endocrine Diseases, Evangelismos Hospital, 106 76 Athens, Greece; mourelatospan@yahoo.com (P.M.); aris_diamad@yahoo.gr (A.D.); 3 Biochemical Department, Evangelismos Hospital, 106 76 Athens, Greece; marypratik@icloud.com; 4 Computational Biomedicine Laboratory, Department of Digital Systems, University of Piraeus, 185 34 Piraeus, Greece; parisgallos@yahoo.com

**Keywords:** long COVID, endothelial biomarkers, DLCO, 6-min walk test, sICAM-1, sVCAM-1

## Abstract

Since the beginning of the pandemic, both COVID-19-associated coagulopathy biomarkers and a plethora of endothelial biomarkers have been proposed and tested as prognostic tools of severity and mortality prediction. As the pandemic is gradually being controlled, attention is now focusing on the long-term sequelae of COVID-19. In the present study, we investigated the role of endothelial activation/dysfunction in long COVID syndrome. This observational study included 68 consecutive long COVID patients and a healthy age and sex-matched control group. In both groups, we measured 13 endothelial biomarkers. Moreover, in the long COVID patients, we evaluated fatigue and dyspnea severity, lung diffusion capacity (DLCO), and the 6-min walk (6MWT) test as measures of functional capacity. Our results showed that markers of endothelial activation/dysfunction were higher in long COVID patients, and that soluble intracellular adhesion molecule 1 (sICAM-1) and soluble vascular adhesion molecule 1 (sVCAM-1) negatively correlated with lung diffusion and functional capacity (sICAM-1 vs. DLCO, r = −0.306, *p* = 0.018; vs. 6MWT, r = −0.263, *p* = 0.044; and sVCAM-1 vs. DLCO, r= −0.346, *p* = 0.008; vs. 6MWT, r = −0.504, *p* < 0.0001). In conclusion, evaluating endothelial biomarkers alongside clinical tests might yield more specific insights into the pathophysiological mechanisms of long COVID manifestations.

## 1. Introduction

In the presence of the still ongoing yet controlled coronavirus disease 2019 (COVID-19) pandemic, numerous studies have been published characterizing and investigating the three principal pathophysiological events of COVID-19: endothelial dysfunction, coagulation, and inflammation [1,2,3]. The wide range of the clinical spectrum of COVID-19 has revealed the need for the development of novel precise and early prediction models that could provide more conclusive information than those that are given by the basic epidemiological and laboratory data [4,5,6,7]. The disruption of the physiological endothelial functions is crucial in COVID-19 progression, which highlights that COVID-19 is a (micro)vascular and endothelial disease [8,9,10,11,12,13,14]. Since the beginning of the pandemic, both COVID-19-associated coagulopathy biomarkers and a plethora of novel endothelial biomarkers have been proposed and tested as prognostic tools of severity and mortality prediction [8,15,16].

Worldwide, patients who have survived COVID-19 have reported experiencing either new onset symptoms after initial recovery from an acute COVID-19 episode or persistent symptoms in the weeks or even the months following the acute infection. Post-COVID-19 condition, or long COVID, as it is commonly known, is defined as symptoms occurring 3 months from the onset of symptoms or the date of the positive test for asymptomatic patients, and lasting for at least 2 months [17], constituting a major public health issue [18]. The reported manifestations are of different intensities and cover a wide range of symptoms, with the most common being fatigue, shortness of breath, and cognitive dysfunction/brain fog, and they generally have an impact on everyday functioning. Greek long COVID patients share the same symptoms [19]. Symptoms might also fluctuate or relapse over time [17]. It is important to note that the presence of the symptoms cannot be attributed to another etiology, and that they are irrespective of the acute infection’s severity. Until now, many studies have examined different aspects of the long COVID condition [20,21], but the underlying mechanism of the pathophysiology of the condition remains unidentified.

Since the involvement of the endothelium has been recognized as a key component in acute infection, it is plausible that it is also involved in long COVID. Evaluating endothelial biomarkers alongside clinical tests may provide more specific insights into the pathophysiological mechanisms of long COVID manifestations [22]. In the present study, we therefore aimed to investigate whether the endothelial biomarkers that have been associated with the acute phase of the infection are also involved in long COVID. Another aim was to examine whether the endothelial biomarkers correlate with long COVID symptoms (namely, fatigue and dyspnea), with lung diffusion capacity, and with the 6-min walk test.

## 2. Materials and Methods

This observational, single-center study included 68 consecutive long COVID patients who presented to the long COVID clinic of the “Evangelismos” Hospital from 31 March 2021 to 21 December 2022 due to persistent symptoms that had lasted at least 2 months. Our sample therefore consisted of different COVID strains, though these were not established with whole genome sequencing. All of the patients who enrolled in the study had a previously confirmed SARS-CoV-2 infection, which had been diagnosed by real-time reverse transcription PCR (RT-PCR) in nasopharyngeal swabs at least 3 months prior. The study was approved by the Hospital’s Research Ethics Committee (112/31-3-2021), and all of the procedures carried out on the patients were in compliance with the Helsinki Declaration. Twenty healthy, pre-pandemic, age- and sex-matched subjects constituted the control group. Healthy subjects were selected from the blood donation department of our hospital. All of them were Caucasian, 9 were men and 11 were women, and the median age of the subjects was 56 (51–58). Informed consent was obtained from all of the subjects involved in the study.

Following study enrolment, demographic characteristics, laboratory data, self-reporting symptoms, and their duration were recorded. The patients were kindly asked to answer a questionnaire with regard to their long COVID symptoms, including the presence or not of fatigue, cough, dyspnea, loss of taste and smell, headaches, arthralgias, diarrhea, abdominal and chest pain, nausea, insomnia, and confusion. Patients were also examined for rashes and alopecia. In order to evaluate fatigue severity, the fatigue assessment scale (FAS) was used [23]. Dyspnea was evaluated with the modified Medical Research Council scale (mMRC) [24].

In all of the patients, functional capacity was evaluated with the 6-mim walk test (6MWT) [25]. Spirometry [26] and single-breath carbon monoxide diffusing capacity (DLCO %pred) [27] were measured (Jaeger MasterScreen™ PFT, CareFusion, Hoechberg, Germany) following international recommendations.

Blood samples were obtained from all of the participants, and the following markers were measured concurrently in samples obtained from patients who presented to the long COVID clinic by enzyme-linked immunosorbent assay (ELISA). The ELISA kits chosen had been previously used and validated in our laboratory [28,29,30]. Circulating levels of thirteen endothelial markers (Table 1) were measured by enzyme-linked immunosorbent assay (ELISA) according to the manufacturers’ instructions.

The assays used two different polyclonal antibodies against the molecules as catching and tagging antibody. The researcher who performed the measurements was blinded to the samples that were measured. Serum ACE activity was determined by the ACE liquid photometric assay (Sentinel Diagnostics, Milan, Italy) on a Cobas c 702 module (Roche Diagnostics International AG, Rotkreuz, Switzerland). The detection limit of the assay is 2.4 U/L.

The data were presented as individual values, mean ± standard deviation (SD) for normally distributed variables, or for variables with a skewed distribution, and the median and interquartile range (IQR) were given. To compare the two groups, either the Student’s *t*-test or the non-parametric Mann–Whitney test was employed. In order to examine the associations between the qualitative variables, the chi-square test was utilized. Correlations were assessed using Spearman’s correlation coefficient. Multiple linear regression analysis was used to model the relationship between DLCO %pred or the 6MWT distance (dependent variables) and sICAM-1, sVCAM-1, age (continuous variables), smoking status, and intensive care unit (ICU) admission (categorical variables). The model was evaluated by the coefficient of determination (R^2^) and the relative *p*-values. All of the statistical tests were performed with a Type I error rate of α = 0.05 and a Type II error rate of β = 0.20 (80% power). The IBM SPSS statistical package, version 22.0 (IBM Software Group, NY, USA), and GraphPad Prism, version 8.0 (GraphPad Software, San Diego, CA, USA), were used for data analysis. All of the *p*-values were calculated based on two-sided tests, and values less than 0.05 were considered statistically significant.

## 3. Results

### 3.1. Patient Characteristics

Table 2 lists the major characteristics of long COVID patients. The median age of the study group was 56 (46–63) years, and 56% of the patients were male. The most frequent comorbidity before hospitalization was hyperlipidemia (19.1%), followed by hypertension (14.7%), diabetes (10.3%), and chronic obstructive pulmonary disease (COPD) (10.3%), while 29.4% were either current smokers or ex-smokers. 

During the acute phase of the disease, the most frequent symptoms were fever (90%), cough (51%), and dyspnea (35%). The majority of the patients were admitted to the ICU (51.5%) and were intubated and mechanically ventilated (47%). A total of 22% had been hospitalized in the ward, while the remaining 26.5% did not require hospitalization. Pneumonia was diagnosed in 72% of the patients during hospital admission. The median length of hospital stay was 33 (19–55) days.

Patients had tested positive for SARS-CoV-2 infection by real-time reverse transcription PCR (RT-PCR) in nasopharyngeal swabs for a median of 139 (86–350) days before visiting the long COVID clinic, and they had been symptomatic for a median of 110 (57–253) days after discharge from the hospital (or from the negative test for outpatients).

### 3.2. Long COVID Symptoms, 6-min Walk Test, and Lung Function Tests

**Figure 1:**
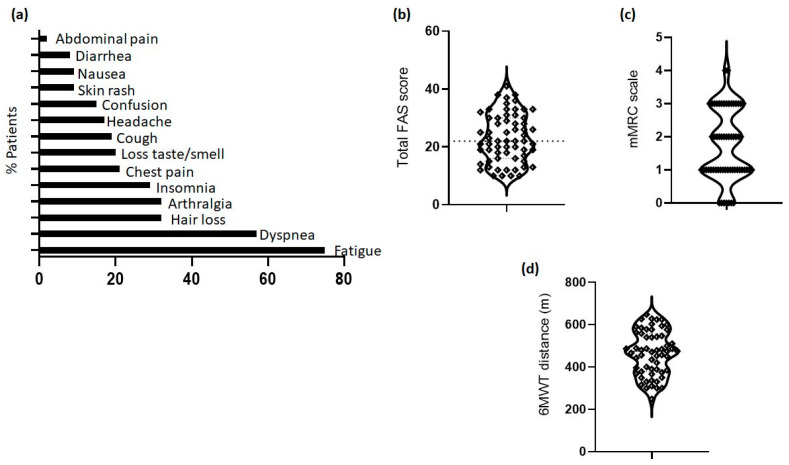
Long COVID symptoms, fatigue, dyspnea severity, and functional capacity. (**a**) Self-reporting symptoms are shown as bars denoting the percentage of patients. (**b**) The fatigue assessment scale (FAS) is shown as a violin plot. FAS scores above the horizontal line (22) denote increased fatigue. (**c**) Dyspnea severity evaluated with the modified Medical Research Council scale (mMRC) and shown as a violin plot. (**d**) Functional capacity evaluated with the 6-min walk test (6MWT). The distance in meters is shown as a violin plot.

Long COVID symptoms included fatigue (N = 62), dyspnea (N = 47), arthralgia (N = 32), hair loss (N = 32), insomnia (N = 29), chest pain (N = 21), loss of taste/smell (N = 20), cough (N = 19), headache (N = 17), confusion (N = 15), skin rash (N = 9), nausea (N = 9), diarrhea (N = 8), and abdominal pain (N = 2) (Figure 1a). Regarding fatigue grading, the median FAS total score was 22 (IQR: 16–30). Although 62 out of the 68 patients reported fatigue as a main symptom, the FAS score was abnormally high (>22) in only 29 patients (42.6%), between 23 and 35 (mild to moderate fatigue) in 24 patients (35.3%), and above 35 (severe fatigue) in 6 patients (8.8%) (Figure 1b). The median value for the mMRC dyspnea scale was 2 (1–3) (Figure 1c). The median 6MWT distance was 474 (378–558) meters (Figure 1d).

**Figure 2:**
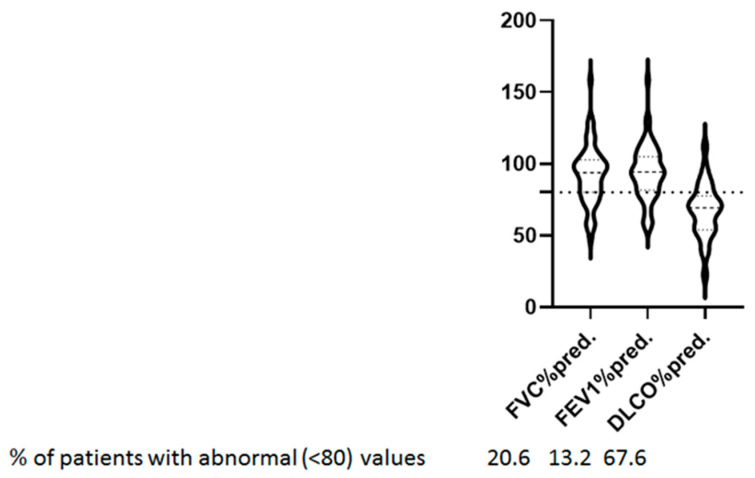
Lung function tests. Forced vital capacity (FVC), forced expiratory volume in 1 s (FEV1), and DLCO are shown as violin plots in 68 long COVID patients. Values below 80% predicted (horizontal line) indicate abnormal measurement.

On average, spirometry was normal. Forced expiratory volume in 1 s (FEV1) was 94.3 (81.6–105.0)%pred., forced vital capacity (FVC) was 93.8 (80.1–102.7)%pred. and FEV1/FVC was 84.0% (81.1–87.4%). Mean DLCO was 67 ± 18.6%pred. DLCO below 80%pred. indicates lung diffusion impairment. Among patients with abnormal DLCO, 38 (55.9%) had a moderate reduction (50–80% pred.) and 8 (11.8%) had a severe reduction (≤50%pred.). Collectively, 47 (69.1%) of the patients had one or more abnormal lung function measurements. Specifically, 9 patients (13.2%) had abnormal FEV1, 14 patients (20.6%) had abnormal FVC, and 46 patients (67.6%) had abnormal DLCO values (% pred.) (Figure 2). 

We have used the lower limit of DLCO (80%) of the predicted value, although the European Respiratory Society recently recommended the use of an approach that is based on z-scores with values below –1.645 to be considered abnormal [31]. However, we still used 80% of the predicted value as the lower limit in order to be consistent with the previous research [32,33].

### 3.3. Levels of Endothelial Biomarkers

**Figure 3:**
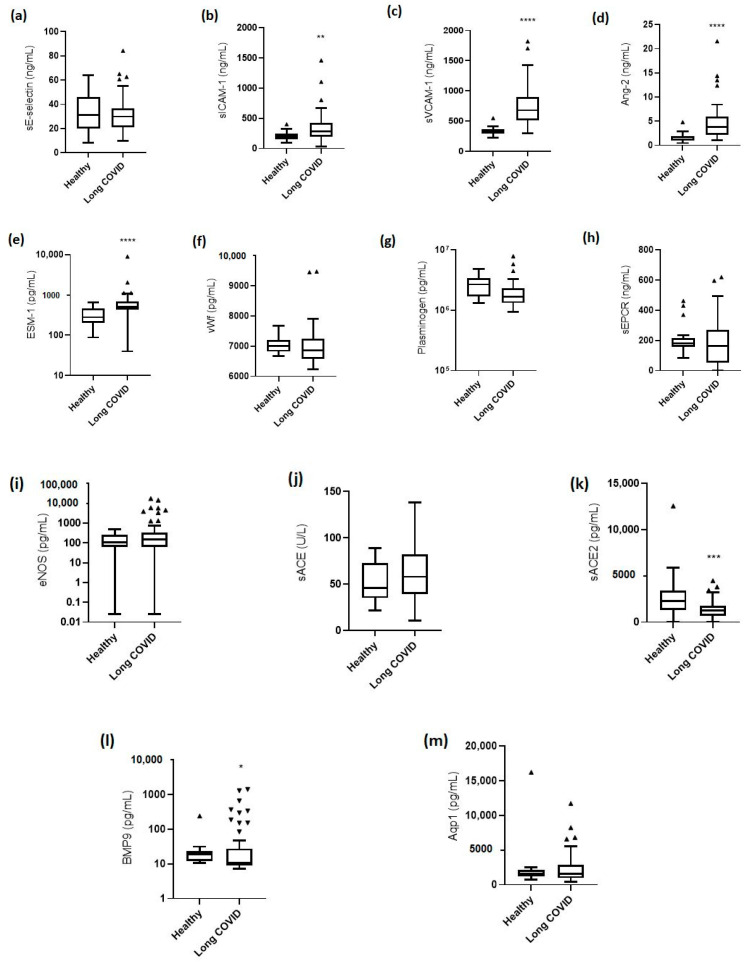
Levels of endothelial biomarkers in healthy controls and long COVID patients: (**a**) soluble (s)E-selectin; (**b**) soluble intercellular adhesion molecule 1 (sICAM-1); (**c**) soluble vascular cell adhesion molecule 1 (sVCAM-1); (**d**) angiopoietin-2 (Ang-2); (**e**) endocan (ESM-1); (**f**) von Willebrand factor (vWf); (**g**) plasminogen; (**h**) soluble endothelial protein C receptor (sEPCR); (**i**) endothelial nitric oxide synthase (eNOS); (**j**) soluble angiotensin-converting enzyme (sACE) activity; (**k**) soluble angiotensin-converting enzyme 2 (sACE2); (**l**) bone-morphogenetic protein 9 (BMP9); and (**m**) aquaporin-1 (Aqp1) levels were measured in 20 healthy controls and 68 long COVID patients. Two-group comparisons were performed with the non-parametric Mann–Whitney test, * *p* < 0.05, ** *p* < 0.01, *** *p* < 0.001; **** *p* < 0.0001. Data are presented as Tukey box plots. Line in the middle, median; box edges, 25th to 75th centiles; whiskers, 75th percentile plus 1.5 times IQR and 25th percentile minus 1.5 times IQR; triangles, outliers.

The levels of the endothelial biomarkers were measured in all 68 long COVID patients and in the healthy control group. Figure 3 shows the levels of endothelial activation/dysfunction in long COVID patients and healthy controls. The levels of sE-selectin were comparable in long COVID patients and the healthy controls [29.9 (21.2–36.7) ng/mL vs. 31.2 (19.8–45.8) ng/mL; *p* = 0.5; Figure 3a]. On the other hand, both cell adhesion molecules, sICAM-1 and sVCAM-1, were elevated in long COVID patients compared to controls [sICAM-1, 286.3 (191.9–428.1) ng/mL vs. 201.2 (158.0–247.6) ng/ml; *p* = 0.002 and sVCAM-1, 676.7 (510.5–899.5) ng/mL vs. 322.3 (289.3–366.8) ng/mL; *p* < 0.0001; Figure 3b,c]. Ang-2 levels were 3.8 (2.2–5.9) ng/mL compared to 1.2 (1.0–1.9) ng/mL in healthy controls (*p* < 0.0001); Figure 3d. ESM-1 levels were 501.4 (426.6–693.9) pg/mL vs. 279.1 (201.8–461.8) pg/mL, *p* < 0.0001; Figure 3e. Indeed, the levels of these biomarkers remained elevated, and were nearly as high as in acute COVID-19 [28,29,30]. Levels of vWf, plasminogen, sEPCR, eNOS, sACE activity, sACE2, BMP9, and Aqp1 in healthy and long COVID patients are shown in Figure 3f–m.

### 3.4. Associations between Endothelial Biomarkers and Long COVID Symptoms, 6-min Walking Test, and Lung Function Tests

Reported symptoms were analyzed with respect to the endothelial biomarkers. The biomarker levels did not differ between the patients reporting symptoms and those that did not. Furthermore, no correlations were found between the total FAS score and the mMRC scale with the various biomarker levels.

**Figure 4:**
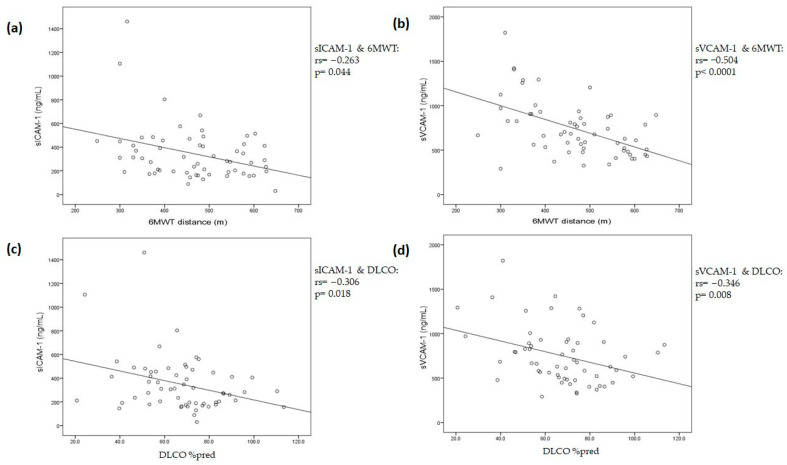
Spearman’s correlation coefficient analysis was performed on sICAM-1 and sVCAM-1 levels and 6MWT and DLCO measurements: (**a**) sICAM-1 and 6MWT (rs = −0.263, *p* = 0.044); (**b**) sVCAM-1 and 6MWT (rs = −0.504, *p* < 0.0001); (**c**) sICAM-1 and DLCO (rs = −0.306, *p* = 0.018); and (**d**) sVCAM-1 and DLCO (rs = −0.346, *p* = 0.008). 6MWT, 6-min walk test; DLCO, carbon monoxide lung diffusing capacity; sICAM-1, soluble intercellular adhesion molecule 1; sVCAM-1, soluble vascular cell adhesion molecule 1.

sICAM-1 and sVCAM-1 showed a negative correlation with 6MWT distance; more specifically, sICAM-1, r = −0.263, *p* = 0.044; Figure 4a and sVCAM-1 r = −0.504, *p* < 0.0001; and Figure 4b. The multiple linear regression model fitted with sICAM-1, sVCAM-1, age, smoking status, and admission to the ICU showed that sICAM-1 and sVCAM-1 could be independently associated with 6MWT distance [−0.126 (−0.221 to −0.030), *p* = 0.011 and −0.121 (−0.205 to −0.038), *p* = 0.005, respectively]. The coefficient of determination (R^2^) of the model was 0.433, with a *p*-value < 0.0001.

The majority of our patients were also characterized by impaired lung diffusion. More specifically, DLCO mean measurement was 67 ± 18.6% predicted. DLCO below 80%pred. was present in 67.6% of the patients, indicating lung diffusion impairment. We were able to find a negative correlation between both cell adhesion molecules, sICAM-1 and sVCAM-1, with DLCO % pred. (sICAM-1, r = −0.306, *p* = 0.018; Figure 4c and sVCAM-1, r = −0.346, *p* = 0.008; Figure 4d). It should be noted that the two molecules did not correlate with each other (*p* = ns). The multiple linear regression model fitted with sICAM-1, sVCAM-1, age, smoking status, and admission to the ICU showed that sICAM-1 and sVCAM-1 were the only variables associated with DLCO measurements [−0.021 (−0.041 to −0.002), *p* = 0.03 and −0.020 (−0.037 to −0.004), *p* = 0.017, respectively]. The coefficient of determination (R^2^) of the model was 0.289, with a *p*-value of 0.003.

## 4. Discussion

In the present study, we found that various markers of endothelial activation/dysfunction and, in particular, sICAM-1, sVCAM-1, Ang-2, ESM-1, sACE2, and BMP9 differed significantly between healthy controls and long COVID patients, with the majority of the latter requiring ICU admission and mechanical ventilation during the acute phase. On the other hand, biomarkers of coagulopathy, inflammation, and vasodilation, including sE-selectin, vWf, plasminogen, sEPCR, eNOS, sACE, and Aqp-1, were comparable to the healthy controls. Moreover, the long COVID population reported a high rate of long COVID symptoms (mostly fatigue), and 68% also had at least mild DLCO abnormalities. We found no correlations between the severity of the reported fatigue and dyspnea and the biomarker levels. However, both sICAM-1 and sVCAM-1 correlated with the DLCO values and the 6MWT. The multiple linear regression analysis fitted with age, smoking status, admission to the ICU, sICAM-1, and sVCAM-1 showed that both CAMs were inversely associated with DLCO measurements and the 6MWT distance.

DLCO is a measurement of the capacity of gas to pass from the alveoli and the capillary endothelium to the red blood cells. DLCO measurement is affected by the surface area and the thickness of the alveolar capillary membrane, the blood volume in the pulmonary capillaries, and the distribution of the inspired gas [34]. In this respect, in a subgroup of patients with pulmonary arterial hypertension, reduced DLCO values were related to the degree of functional capillary surface area loss [35]. Abnormal functional capacity and lung function tests, including <80%pred DLCO, have been reported in COVID-19 survivors, and this is indicative of continued damage in the alveolar-capillary space and possible ongoing fibrosis [36]. In a cohort of 101 post-COVID-19 patients, with the majority (73%) recovering from severe pneumonia, six weeks post-discharge, the mean DLCO% pred. was 70.3%, which was a value similar to the baseline percentage of the cohort in the present study [37]. Moreover, even though only the presence of severe/critical disease could predict DLCO reduction [38] in multivariate models, in our study, sICAM-1 and sVCAM-1 were inversely correlated with % pred. DLCO independently of ICU admission. In another study, these abnormalities were also associated with elevated plasma levels of epithelial and endothelial cell markers, namely, surfactant proteins, sICAM-1, and Ang-2 [39]. Our results partly agree with these findings; we also found significantly elevated Ang-2 levels compared to the healthy controls. However, Ang-2 levels did not seem to correlate with the presence of dyspnea or its severity (mMRC scale) or with reduced lung diffusing capacity. The endothelial biomarkers that correlated with both 6MWT and DLCO values were sICAM-1 and sVCAM-1. It may thus be suggested that sICAM-1 and sVCAM-1 levels represent persisting endothelial lung damage, which, according to data from other studies, return to normal levels with elapsing time, even after several months [40,41,42,43]. Ang-2 may play a less important role in ongoing lung damage, and this is also supported by the previous literature [44].

Even after discharge from rehabilitation, post-COVID-19 patients suffer from a significant reduction in physical function, exercise capacity, and the ability to perform daily activities [45]. An impaired functional capacity, measured with the 6MWT, which is one of the most widely used exercise field tests, is a common finding. Patients with severe/critical COVID-19 shortly after discharge (median 18 days) were characterized by severely impaired 6MWT distance (median 344 m) [46]. Patients in the present study could not reach the average 6MWT distance (659 ± 62 m), as determined in healthy subjects of a similar age (55–75), more than 4 months after disease onset [47]. Τo the best of our knowledge, this is the first study to show a correlation between an impaired 6MWT distance and elevated endothelial damage markers in long COVID patients independently of disease severity (ICU admission).

We have also reported deranged serum levels for some endothelial biomarkers that have not previously been considered abnormal in long COVID patients. Studies until now, indeed, have shown that ESM-1 is not involved in long COVID [44,48]. In our study, however, ESM-1 levels were increased compared to healthy controls, yet these levels seemed to be reduced compared to the acute phase of the disease [28], and this is indicative of persisting endothelial lung damage. In the same manner, a study reported similar levels of sACE2 in both long COVID patients and normal controls [44]. However, in our long COVID cohort, sACE2 levels were lower than in the healthy controls. Finally, reduced circulating BMP9 levels, a pulmonary endothelial-protective factor, have been described in hospitalized COVID-19 patients, and this has prompted the authors of this study to propose that BMP9 could offer a novel approach to preventing increased pulmonary endothelial permeability [49]. In our study, BMP9 was also decreased in long COVID patients compared to the controls, which indicates impaired endothelial protection. Since both BMP9 and ACE2, which are known for their reparative role in the endothelium, were found downregulated in the long COVID group, an ongoing endotheliopathy in long COVID syndrome can be theorized, mostly affecting the endothelial integrity and its immune functions, with concomitant dysregulation of the reparative mechanisms of endothelial permeability [49,50].

In our study, several biomarkers that have previously been shown to have higher serum levels in long COVID syndrome were found to be comparable to those of the normal controls. We were not able to find differences in sE-selectin levels between the long COVID patients and the healthy controls, nor did we find a correlation with lung diffusion. This finding, in combination with the high levels of sE-selectin reported during the acute phase [29,51], may suggest that sE-selectin could have a particular role in the initial phase of the pulmonary vasculature damage. Similarly, in our long COVID patients, coagulation and fibrinolysis seemed to return to normal. More specifically, vWf levels returned to the control values, while plasminogen levels increased and were comparable to controls. Studies up until ours have shown higher levels of vWf in long COVID patients compared to controls [40,52,53], which suggests a hypercoagulability state up to 1 year after acute infection [40]. In our long COVID cohort, soluble eNOS levels were also comparable to healthy controls, suggesting restoration of synthesis of the vasodilator, nitric oxide. Finally, serum levels of Aqp1, which is a molecule expressed in alveolar epithelial cells and microvascular endothelial cells, and has been found to increase in patients with COVID-19 compared to healthy controls [54], were normalized in our long COVID population.

The limitations of our study should be stated. Firstly, it was a single-center study that included a relatively small number of long COVID patients, and, therefore, the risk of underestimating existing correlations of biomarkers with both DLCO and long COVID symptoms might be high. Despite this, we were able to show significant correlations between DLCO measurements and the 6-min walk test distance with the levels of specific endothelial biomarkers. Secondly, we did not measure lung volumes and we did not perform lung imaging studies, particularly high-resolution chest computerized tomography. Thirdly, we did not report consecutive measurements or measurements prior to acute COVID-19, disease lung function, and/or imaging studies, which could have allowed us to exclude previously existing respiratory diseases that are not attributed to long COVID. Lastly, we did not compare our findings with COVID-19 patients who did not develop long COVID. However, we were able to assess the endothelium globally by measuring 13 serum biomarkers, which reflects most of the aspects of systemic and pulmonary endothelial function. More research is needed in order to investigate the damage that different COVID-19 strains are causing to the endothelium.

## 5. Conclusions

In the present study, we showed that in long COVID patients, lung function test abnormalities, including impaired diffusion, are common. Increased levels of Ang-2, endocan, sICAM-1, and sVCAM-1 as well as reduced levels of the endothelial protective markers BMP9 and sACE2 are present compared to normal age and sex-matched controls, and this supports ongoing endothelial activation and impaired endothelial reparative mechanisms. These molecules could be attractive diagnostic and therapeutic targets. The levels of these biomarkers were not found to be associated with the presence or the severity of long COVID symptoms, but sICAM-1 and sVCAM-1 were independently associated with reduced DLCO and the 6MWT distance. Among the several hypothesized mechanisms for long COVID pathogenesis, including immune dysregulation, dysfunctional neurological signaling, autoimmunity, clotting, and endothelial abnormality seem to be cornerstone mechanisms of impaired respiratory function. These findings warrant further investigation in order to clarify their clinical importance and time evolution so as to prevent (if possible) and treat these sequalae.

## Figures and Tables

**Table 1:** Detection limits of measured endothelial biomarkers.

Endothelial Biomarkers	Intra-Assay Coefficient of Variability (CV) (%)	Detection Limit	Manufacturer
sE-selectin	5.9	0.027 ng/mL	R&D Systems Inc., Thermofisher scientific, Waltham, MA, USA
Ang-2	5.9	21.3 pg/mL	R&D Systems Inc., Thermofisher scientific, Waltham, MA, USA
sICAM-1	4.6	0.254 ng/mL	R&D Systems Inc., Thermofisher scientific, Waltham, MA, USA
sVCAM-1	3.1	1.26 ng/mL	R&D Systems Inc., Thermofisher scientific, Waltham, MA, USA
sEPCR	5.8	0.3 ng/mL	R&D Systems Inc., Thermofisher scientific, Waltham, MA, USA
eNOS	4.2	25 pg/mL	R&D Systems Inc., Thermofisher scientific, Waltham, MA, USA
Plasminogen	<8	46.875 pg/mL	Fine Biotech Co., Wuhan, China
ESM-1	6.3	<10 pg/mL	OriGene Technologies, Inc., Rockville, MD, USA
vWf	5.2	<50 pg/mL	OriGene Technologies, Inc., Rockville, MD, USA
sACE2	4.8	<10 pg/mL	OriGene Technologies, Inc., Rockville, MD, USA
BMP9	4.8	<10 pg/mL	OriGene Technologies, Inc., Rockville, MD, USA
Aqp1	4.5	75 pg/mL	Assay Genie, Dublin, Ireland
sACE	1.4	2.4 U/L	Sentinel Diagnostics, Milan, Italy

Definition of abbreviations: Ang-2, angiopoietin 2; Aqp1, aquaporin-1; BMP9, bone-morphogenetic protein 9; eNOS, endothelial nitric oxide synthesase; ESM-1, endothelial cell-specific molecule 1; sACE = soluble angiotensin-converting enzyme; sACE2 = soluble angiotensin-converting enzyme 2; sEPCR, soluble endothelial protein C receptor; sICAM-1, soluble intercellular adhesion molecule 1; sVCAM-1, soluble vascular cell adhesion molecule 1; vWf, von Willebrand factor.

**Table 2:** Demographics, clinical characteristics, severity of symptoms, and endothelial biomarkers of long COVID patients.

Characteristics	Long COVID Patients
Number of patients, N	68
Age (years), (median, IQR)	56 (46–63)
Sex, N (%)	
Male	38 (55.9%)
Female	30 (44.1%)
Days after positive-test, (median, IQR)	139 (86–350)
Hospitalized, N (%)	
ICU	35 (51.5%)
Mechanical ventilation	32 (91.4%)
High-flow oxygen therapy	3 (8.6%)
Ward	15 (22.0%)
Outpatients	18 (26.5%)
Hospitalization days, (median, IQR)	33 (19–55)
BMI (kg/m^2^), (median, IQR)	29.0 (24.9–33.1)
Smoking/Ex-smoking habit, N (%)	
Yes	20 (29.4%)
No	48 (70.6%)
Comorbidities, N (%)	32 (47.1%)
Hyperlipidemia	13
Hypertension	10
Diabetes	7
COPD	7
Coronary artery disease	3
Chronic renal failure	1
Symptoms during acute COVID-19, N (%)	
Fever	61 (89.7%)
Cough	35 (51.4%)
Dyspnea	24 (35.3%)
Fatigue	20 (29.4%)
Arthralgia/Myalgia	17 (25.0%)
Loss of taste/smell	15 (22.1%)
Diarrhea	14 (20.6%)
Headache	14 (20.6%)
**Laboratory data**	
CRP (mg/dL), (median, IQR)	0.2 (0.1–0.3)
D-dimers (µg/mL), (median, IQR)	0.32 (0.24–0.57)
LDH (U/L), (median, IQR)	183 ± 36
Creatinine (mg/dL), (median, IQR)	0.8 (0.7–0.9)
Platelets (per μL), (median, IQR)	241,000 (207,000–270,000
White blood cell count (per μL), (mean ± SD)	6116 ± 1615
Neutrophils (%), (mean ± SD)	55.5 ± 7.9
Lymphocytes (%), (mean ± SD)	34.4 ± 7.4
**Fatigue, dyspnea, 6-min walk, diffusion capacity, and spirometry**	
FAS scale, (median, IQR)	22 (16–30)
mMRC dyspnea scale (median, IQR)	2 (1–3)
6MWT (meters), (median, IQR)	474 (378–558)
DLCO %pred., (mean ± SD)	67 ± 18.6%
FEV1 %pred., (median, IQR)	94.3 (81.6–105.0)
FVC %pred., (median, IQR)	93.8 (80.1–102.7)
FEV-1/FVC (median, IQR)	84.0 (81.1–87.4)
**Endothelial biomarkers**	
sE-selectin (ng/mL), (median, IQR)	29.9 (21.2–36.7)
sICAM-1 (ng/mL), (median, IQR)	286.3 (191.9–428.1)
sVCAM-1 (ng/mL), (median, IQR)	676.1 (507.6–901.0)
Ang-2 (ng/mL), (median, IQR)	3.8 (2.2–5.9)
ESM-1 (pg/mL), (median, IQR)	501.4 (426.6–693.9)
vWf (pg/mL), (median, IQR)	6862 (6574–7241)
Plasminogen (pg/mL), (median, IQR)	1,680,182 (1,312,186–2,309,512)
sEPCR (ng/mL), (median, IQR)	164.8 (54.2–268.5)
eNOS (pg/mL), (median, IQR)	149.8 (62.9–338.0)
sACE (U/l), (median, IQR)	58 (39–83)
sACE2 (pg/mL), (median, IQR)	1219 (661–1710)
BMP9 (pg/mL), (median, IQR)	10.6 (8.8–27.8)
Aqp1 (pg/mL), (median, IQR)	1622 (956–2837)

Data are presented as number of patients (N), percentages of total related variable (%), and mean ± SD for normally distributed variables or median (IQR) for skewed data. Definition of abbreviations: 6MWT, 6-min walk test; Ang-2, angiopoietin 2; Aqp1, aquaporin-1; BMI, bone mass index; BMP9, bone-morphogenetic protein 9; COPD, chronic obstructive pulmonary disease; CRP, C-reactive protein; DLCO, carbon monoxide lung diffusing capacity; eNOS, endothelial nitric oxide synthesase; ESM-1, endothelial cell-specific molecule 1; FAS, fatigue assessment scale; FEV1, forced expiratory volume in 1 s; FVC, forced vital capacity; ICU, intensive care unit; mMRC; modified Medical Research Council; LDH, lactate dehydrogenase; sACE = soluble angiotensin-converting enzyme; sACE2 = soluble angiotensin-converting enzyme 2; sEPCR, soluble endothelial protein C receptor; sICAM-1, soluble intercellular adhesion molecule 1; sVCAM-1, soluble vascular cell adhesion molecule 1; vWf, von Willebrand factor.

## Data Availability

The data presented in this study are available on request from the corresponding author. The data are not publicly available due to restrictions (privacy and ethical).
